# Dyadic Adjustment of Couples and State Anxiety in Patients Tested for Sexually Transmitted Infections

**DOI:** 10.3390/jcm13051449

**Published:** 2024-03-02

**Authors:** Martina-Luciana Pintea-Trifu, Mihaela-Laura Vică, Daniel-Corneliu Leucuța, Horia George Coman, Bogdan Nemeș, Horea-Vladi Matei

**Affiliations:** 1 Department of Cellular and Molecular Biology, Iuliu Hațieganu University of Medicine and Pharmacy, 400349 Cluj-Napoca, Romania; pintea.trifu.martina@elearn.umfcluj.ro (M.-L.P.-T.); mvica@umfcluj.ro (M.-L.V.); hmatei@umfcluj.ro (H.-V.M.); 2 Department of Medical Informatics and Biostatistics, Iuliu Hațieganu University of Medicine and Pharmacy, 400349 Cluj-Napoca, Romania; 3 Department of Medical Psychology and Psychiatry, Iuliu Hațieganu University of Medicine and Pharmacy, 400012 Cluj-Napoca, Romania; hcoman@umfcluj.ro (H.G.C.); nemes.bogdan@umfcluj.ro (B.N.)

**Keywords:** sexually transmitted diseases, reproductive health, anxiety, couples, venereology, adjustment, young adults, test anxiety scales, bacterial sexually transmitted disease

## Abstract

**Background**: While existing literature addresses the psychological impact of HIV, there is a notable gap in data regarding other sexually transmitted infections (STIs). This study aims to fill this gap by evaluating the association between STIs, the psychological profile of patients as measured by anxiety levels, and the impact on couple adaptability. **Methods**: A prospective investigation was conducted in Romania, from November 2021, including individuals with high suspicion of STI and healthy controls. Data collection comprised a questionnaire, the Dyadic Adjustment Scale (DAS), and State-Trait Anxiety Inventory (STAI Y-1). Statistical methods, including multivariate logistic and linear regressions, were used to carry out the analyses. **Results**: The participant cohort consisted of 441 individuals. STI participants exhibited consistently lower DAS scores, notably in dyadic adaptability (DA) (*p* = 0.031), dyadic satisfaction (DS) (*p* = 0.006), and affectional expression (AE) (*p* = 0.016). Multivariate logistic regression with adjustment for confounders confirmed a significant association between STIs and atypical DAS responses (2.56-fold increase). STAI T scores were significantly higher in the STI suspected group (*p* < 0.01), remaining robust after adjusting for confounders in a multiple linear regression model. **Conclusions**: Our prospectively designed study highlights the mental health repercussions associated with STIs. This is evident through the diminished DAS scores and heightened STAI Y-1 scores observed in individuals with suspected STIs.

## 1. Introduction

Sexually transmitted infections (STI) are among the most common infectious diseases in the world, with an average of 1 million newly diagnosed cases every day, having a major impact on sexual health, reproductive health, and mental health [1]. Variations in incidence primarily stem from socioeconomic factors, cultural and moral attitudes, as well as the influences of travel and globalization [2,3,4]. Over the past 15 years, the successful implementation of HIV antiretroviral therapy has been associated with a rise in risk behaviors and STIs on a global scale [5]. In 2016, the worldwide prevalence rates for women were reported as follows: 3.8% for chlamydia (with a 95% uncertainty interval, UI, of 3.3–4.5) and 0.9% for gonorrhea (95% UI: 0.7–1.1) [6]. For men, the prevalence rates were 2.7% for chlamydia (95% UI: 1.9–3.7) and 0.7% for gonorrhea (95% UI: 0.5–1.1) [6]. The highest prevalence of the mentioned STIs concerns the 20–24 age group, with *Chlamydia trachomatis* being more frequent in women and *Neisseria gonorrhoeae* being more frequent in men [7].

Considering the high incidence of STIs and their economic burden on the medical systems [8], it is important to determine the relationship between STIs and anxiety in the context of the disease, both for the individual patient and their couple relationship. In the specialized literature, numerous studies address the psychological impact of human immunodeficiency viruses (HIV), yet there are insufficient data on other STIs. A research investigation on Patient-Delivered Partner Therapy, involving the provision of medication to partners of patients diagnosed with bacterial STIs without formal consultation or counselling, revealed that nearly one third of the participants experienced feelings of anxiety, concern about infidelity, and embarrassment. Moreover, the study found that women were 2.43 times more likely than men to experience such worries. Anxiety related to potential partner violence among patients undergoing testing for bacterial STIs was further observed in specific circumstances, including the absence of prior STI diagnosis and lack of medical insurance coverage [9]. Physical, psychological, and sexual intimate partner violence were also reported among men who have sex with men and transgender women, particularly within stable partnerships and often in conjunction with substance use [10]. In a separate investigation involving a young cohort from Canada, it was discovered that undergoing testing for STIs can induce feelings of anxiety or stigma. The study revealed that while online STI testing may mitigate external stigma, it does not alleviate internalized stigma or the associated feelings of guilt and anxiety [11]. Besides stigma and anxiety, knowledge was shown to have an effect on STI testing intention [12]. A cross-sectional analysis of the National Ambulatory Medical Care Survey data from the Centers for Disease Control and Prevention spanning the years 2009 to 2016 evaluated the prevalence of sexually transmitted infection (STI) testing in outpatient physician offices. The results indicated a suboptimal level of STI testing, falling below the recommended threshold, with elevated rates observed among female patients and individuals of non-Hispanic black ethnicity, followed by Hispanic patients [13].

The importance of investigating the relationship between STIs and the psychological profile of patients is particularly emphasized, considering studies that have identified psychological or psycho-social interventions to reduce at-risk sexual behaviors among specific vulnerable groups [14,15,16]. High-intensity (more than 2 h) interventions decreased STI incidence among adolescents and adult populations [17]. Hence, examining the link between sexually transmitted infections (STIs) and anxiety, as well as couple adaptability, contributes to the development of psychological interventions. This research aligns with the World Health Organization’s 2030 Agenda, specifically objective 3.7, which focuses on enhancing sexual and reproductive health [18].

The state of anxiety has been studied through the examination of the following emotional states: tension, stress, unease, apprehension, fright, nervousness, agitation, indecision, and disturbance. Adaptability in couples (both in married couples and those in cohabiting relationships) has not been previously studied in couples with STIs of bacterial etiology. However, understanding adaptability is crucial in comprehending the impact of these pathologies on the quality of life of patients and their compliance with testing and treatment.

The objectives of this study were to assess the relationship between the psychological profile of the patient and the presence/suspicion of STIs, specifically to observe the association between STIs and the level of anxiety in the context of the disease and the impact of the pathology on couple adaptability.

## 2. Materials and Methods

### 2.1. Study Design and Setting

This prospective case–control study was conducted in the north-west region of Romania, encompassing patients seeking medical services in dermatology, obstetrics-gynecology, urology, and general practice across the geographical expanse of the region, including the counties of Cluj (566,475 inhabitants) [19] and Bistrița-Năsăud (303,559 inhabitants) [19] (“Iuliu Hațieganu” University of Medicine and Pharmacy Cluj-Napoca, County Emergency Hospital Cluj-Napoca, County Emergency Hospital Bistrița, Sanovil Clinic Bistrița, Bistrița Sanitary Theoretical High-School (Post Secondary School), Neuropsychiatric Recovery and Rehabilitation Center for Youth Beclean). The STI epidemiology literature in these counties is limited. One study found the most common bacterial STI as *CT* (present in 22% of the positive enrolled patients), followed by *Ureaplasma urealyticum* (9%), and *NG* (7%) [20]. The current practice in public hospitals does not use PCR tests for bacterial STIs, and there are no data on the practices in the private sector regarding rates of testing. Notably, the participants’ residences extended beyond these counties. A consecutive sampling recruitment was used within medical services in dermatology, obstetrics-gynecology and urology. For the other patient sources, convenience sampling was carried out. The data collection started in November 2021, spanning both the SARS-CoV-2 pandemic and the post-pandemic periods in Romania up to November 2023. The study group included patients with a high suspicion of STI, and the control group included healthy subjects.

For data and biological sample collection, the study participants had to sign an informed consent form, explicitly informing them that information would be gathered from questionnaires and urine, urethral, or vaginal secretion samples. All these forms and working protocols have received approval from the Ethics Committee of the “Iuliu Hațieganu” University of Medicine and Pharmacy in Cluj-Napoca, as well as the ethics committees of the public and private institutions where the study took place (“Iuliu Hațieganu” University of Medicine and Pharmacy Cluj-Napoca—code AVZ10 from 8 November 2021, Bistrița Sanitary Theoretical High-School (Postsecondary School)—code 2531 from 15 November 2021, Neuropsychiatric Recovery and Rehabilitation Center for Youth Beclean—code 4939 from 22 November 2021, County Emergency Hospital Cluj-Napoca—code 4980 from 10 February 2022).

All personal data of the enrolled subjects and their processing adhere to EU Regulation no. 679/2016 (concerning the protection of individuals regarding the processing of personal data and the free movement of such data), a regulation that came into force in Romania on 25 May 2018.

Patient information, handled in compliance with the GDPR law, was comprehensive and covered a spectrum of factors: general patient data, demographic details, source of income, sexual history, environmental background, STI antecedents, symptomatology, comorbidities, sexual orientations, the presence of partners with STIs, the number of sexual partners, engagement with new or simultaneous partners, age at sexual debut, methods employed for protection against STIs, and the use of toxic substances. Additionally, the study incorporated the application of assessment tools, specifically the S-Anxiety Scale (STAI Form Y-1) and the Dyadic Adjustment Scale (DAS). The psychotherapist who utilized the tools was licensed to use both psychological instruments. Both tools have been validated for the Romanian population, and their standardization was applied specifically for use within this demographic. The timeframe of data collection ensured a thorough exploration of the impact of the SARS-CoV-2 pandemic on the variables under investigation.

### 2.2. Participants

Inclusion criteria for the study subjects (included in the STI suspected group) encompassed adults (over 18 years) presenting at a medical service for symptoms suggestive of a bacterial STI (such as *Chlamydia trachomatis*, *Neisseria gonorrhoeae*, *Mycoplasma genitalium*, and other similar pathogens), sexual partners of patients infected with a sexually transmitted pathogen, and informed consent for study participation. The diagnosis was performed by a physician using patient history and general objective examination. The criteria for bacterial STI included the following: dysuria, pain in the lower abdomen, dyspareunia, genital discharge, metrorrhagia, genital itching, and macules, papules, vesicles or pustules on an erythematous background, or discontinuity lesions upon objective examination. The assessment of sexual partners of patients infected with a sexually transmitted pathogen was performed by means of the questionnaire. Exclusion criteria comprised the absence of characteristic symptoms of sexually transmitted diseases, lack of a partner with a sexually transmitted disease, and refusal to participate in the study. The inclusion criteria for the control group were as follows: adults (over 18 years old), not hospitalized, with no symptoms suggestive of a bacterial STI (assessed by the questionnaire), and those who have not recently presented to a medical service for symptoms specific to an STI, from the same geographical area, the same age group, and distribution in the environment of origin as the patients in the study group, who have signed the informed consent form for study participation. Exclusion criteria for the control group were hospitalization at the time of enrollment in the study, genitourinary problems, and other health problems.

### 2.3. Variables and Measurement

The data collected from subjects were obtained through questionnaires completed by the participants in the investigator’s presence, featuring multiple-choice responses for all questions, except age and age at the onset of sexual activity (where subjects were required to provide numerical responses). For gender, response options included female, male, and other; for the origin environment, options included urban and rural; for income source, options included employed, self-employed, pensioner/veteran/revolutionary/person with a disability, student, social assistance, unemployed, without occupation, and other categories; for substances, possible responses were smoking, alcohol, psychoactive substances/drugs, or none; for symptoms, participants could choose from pain during urination, lower abdominal pain, genital discharge, pain during sexual intercourse, bleeding between menstruations, genital itching; for comorbidities, possible responses included pelvic inflammatory disease, cervicitis/salpingitis/endometritis, human papilloma virus infection, infertility, history of ectopic pregnancies, prostatitis/epididymitis, arthritis; the three options for sexual orientation were heterosexual, homosexual, or other; for means of protection against sexually transmitted diseases, the three response options were yes, always, occasionally, and no. Questions with yes or no answers included whether they had a partner with an STI, if they had more than two sexual partners, if they had more than two sexual partners during the SARS-CoV-2 pandemic if they had a new sexual partner, and if they had simultaneous sexual partners (no period specification).

DAS measures the quality of adaptation between partners in marital or consensual dyadic relationships. It is an assessment tool consisting of 32 items, including four subscales: Dyadic Consensus (DC), Dyadic Satisfaction (DS), Affectional Expression (AE), and Dyadic Cohesion (DH). Additionally, a total adaptation score (DA) is calculated by summing the scores of the four subscales. Scores from the profile forms are reported in T-scores. T-scores are standardized scores with the useful feature that each subscale has the same mean and standard deviation.

T-scores are divided into the following categories: <34 (moderately atypical—indicates a significant problem), 35–39 (mildly atypical—indicates a significant problem), 40–44 (slightly atypical—borderline, possible reason for concern), 45–55 (average, normal scores, no reasons for concern), 56–60 (slightly atypical), 61–65 (mildly atypical), 66–70 (moderately atypical) and >70 (markedly atypical). The classification was created and validated by the authors of the scale, and it is written in the scale application guide. These categories are established to provide a structured framework for interpreting the T-scores obtained from DAS. They help clinicians and researchers understand the level of relationship functioning indicated by the scores. This classification system allows for clearer identification of potential areas of concern or strength within the relationship, guiding appropriate interventions or further evaluation as needed.

DC assesses the degree of understanding between partners regarding important relationship factors such as money, religion, recreational activities, friends, household duties, and time spent together. DS measures the level of tension and frustration in the relationship and the level at which the individual considers the relationship ended. AE measures the person’s satisfaction with the expression of affection and sexuality in the relationship. DH assesses common interests and activities shared by the couple [21].

The S-Anxiety Scale (STAI Form Y-1) consists of 20 statements assessing how the respondents feel right now, at this moment. The inventory measures anxiety as a state, focusing on feelings of distrust, tension, nervousness, and worry. Scores range from a minimum of 20 to a maximum of 80, with higher scores indicating the presence of a high level of anxiety. However, in research, T-scores are recommended. These represent linearly transformed, normalized versions of raw scores, standardized to have a mean of 50 and a standard deviation of 10. For subjects with T-scores above 60–65, increased attention is recommended, along with conducting an in-depth interview focusing on the symptoms experienced by patients related to anxiety [22].

### 2.4. Statistical Analyses

Categorical data were presented as counts and percentages. Skewed continuous variables were presented as medians and interquartile ranges. Comparisons between the two groups concerning qualitative characteristics were made using the chi-squared test or Fisher exact test. Comparisons between the two groups concerning skewed continuous variables were performed with the Wilcoxon rank-sum test. The 95% confidence intervals for the difference between two groups associated to the Wilcoxon rank-sum test were computed using non-parametric methods. We developed two multivariate logistic regression models to predict atypical responses suggestive of issues from the perspective of DAS DA. In both models, the primary variable of interest was the group (STI vs. control). The first model incorporated adjustments for age, gender, and exposure to various toxic substances (including either alcohol, psychoactive substances, or smoking). We chose four variables in this model, using the rule of ten subjects per variable, out of the smallest category of the dependent variable (in this case with 41 subjects), to prevent overfitting. The second model controlled for the toxic substances separately and explicitly but with an increase in overfitting. For both models, the Hosmer and Lemeshow test was used to assess the goodness of fit, and the variance inflation factor was used to assess the multicollinearity. We built a linear regression model to predict the STAI T score having the group (STIs vs. control) as a predictor of interest and adjusted for age, sex, place of residence, alcohol consumption, psychoactive substance use, smoking, and income source. The odds ratio, 95% confidence intervals (CI), and *p*-values were reported for each model. The following assumptions of the model were checked: the normality of the residuals (with a quantile-quantile plot), the presence of heteroskedasticity (with the Breusch Pagan test), the presence of multicollinearity (with the variance inflation factor and correlations), the linearity of the predictors with the dependent variable (with component residual plots). The model coefficients, 95% CI, and *p*-values were reported for each variable. For all statistical tests, a two-tailed *p*-value was reported, and a 0.05 significance level was used. All statistical analyses were performed with the R environment for statistical computing and graphics (R Foundation for Statistical Computing, Vienna, Austria), version 4.3.1 [23].

## 3. Results

In the present study, the participant cohort consisted of 441 individuals (244 patients in the STI suspected group and 197 individuals in the control group). The median age within this cohort was identified as 32 years, with an interquartile range spanning from 21 to 42 years. The age range of the participants extended from 18 to 74 years. Notably, the sample exhibited a predominance of female participants, constituting 81.6% of the total (equating to 360 individuals). Detailed demographic and clinical characteristics of the participants, divided into two groups—those with suspicion of STIs and the control group—are comprehensively presented in Table 1.

The median age was significantly higher in the STI suspected group compared to the control group (*p* < 0.001). There was no statistically significant difference concerning the sex or rural background of the participants in the two groups (*p* = 0.392). A significantly higher percentage of individuals with STIs had a history of STIs compared to the control group (*p* < 0.001). All reported symptoms (dysuria, lower abdominal pain, dyspareunia, genital discharge, metrorrhagia, genital itching) were significantly more common in the STI suspected group (*p* < 0.001). Incidences of pelvic inflammatory disease, cervicitis/salpingitis/endometritis, HPV infection, and sterility were significantly higher (*p* values between 0.012 and 0.026) in the STI suspected group. The majority were heterosexual in both groups, with no significance (*p* = 0.392). Individuals in the STI suspected group were statistically significant more likely to have a partner with a STI compared to the control group (*p* = 0.001). No significant difference was found in the number of people having more than two sexual partners in general (*p* = 0.117), but during the pandemic period (*p* = 0.006), the STI suspected group reported significantly more multiple partners. More individuals in the STI suspected group reported new sexual partners compared to the control group, with *p* < 0.001. The median age at sexual debut was the same in both groups (*p* = 0.942). The STI suspected group reported significantly less consistent use of protection against STIs (*p* = 0.003). No significant differences were observed in alcohol (*p* = 0.468) or tobacco use (*p* = 0.129); however, psychoactive drug use (*p* = 0.059) was higher in the STI suspected group, nearing significance.

The investigation explored the presence of any disparities between participants in the STI suspected group and those in the control group in relation to their scores on the DAS and STAI S-anxiety assessments, as detailed in Table 2. Analysis revealed that the STIs group exhibited lower median scores across all subscales of the DAS, compared to the control group. This observation was particularly pronounced and statistically significant in the total DAS (DA) score (*p* = 0.031), as well as in the DAS (DS) (*p* = 0.006) and (AE) (*p* = 0.016) subscales. Further categorization of the DAS DA total score also demonstrated statistically significant differences between the two groups (*p* = 0.025). In contrast, both the STAI raw scores (*p* < 0.001) and STAI T scores (*p* < 0.001) were notably and statistically significantly higher in the STIs group relative to the control group.

To delve deeper into the association between the DAS scores and STI or control group belonging, we developed two multivariate logistic regression models. These models aimed to predict atypical responses suggestive of issues from the perspective of DAS DA, as outlined in Table 3. In both models, the primary variable of interest was the presence of an STI. The first model incorporated adjustments for age, gender, and exposure to various toxic substances (including either alcohol, psychoactive substances, or smoking). This model prevented overfitting. The findings indicated that, even after accounting for these confounders, the association between DAS DA scores and STIs persisted as statistically significant (*p* = 0.016). Specifically, having an STI was associated with a 2.56-fold increase in the odds of exhibiting an atypical response on the DAS DA scale. The second model differed in its methodology, as it separately included each toxic substance as a confounder, albeit at the risk of overfitting. Despite this, the link between DAS DA scores and STIs remained statistically significant in this model as well (*p* = 0.016). The predictive accuracy of these models, evaluated using the area under the receiver operating characteristic curve, was 68.31 (with a 95% CI of 59.15–77.47) for the first model and marginally higher at 69.03 (with a 95% CI of 60.44–77.62) for the second model.

To verify the robustness of the association between the STAI T score and STI suspected group, we built a multiple linear regression model predicting the STAI T score based on STI and adjusted for age, sex, place of residence, alcohol consumption, psychoactive substance use, smoking, and income source (Table 4). Even after adjustment for confounders, the relation between STIs and STAI T score remained statistically significant, with STI suspicion increasing the STAI T score (*p* = 0.021).

## 4. Discussion

Our study, using a prospective design, revealed novel insights into mental health implications associated with STIs. The most notable outcomes include statistically significant differences in psychological profiles between the STI suspected group and the control group. Specifically, individuals within the STI group demonstrated lower scores in the DAS assessment, highlighting a lower adaptability in couples where at least one partner harbors suspicion of an STI. Furthermore, this group exhibited significantly higher scores on the STAI scale, indicating a heightened level of anxiety at the moment.

The total scores on the DAS within the study group exhibited a statistically significant decrease compared to the control group. This suggests that either suspicion of an STI in one of the partners has resulted in the deterioration of the relationship or a pre-existing low adaptability and mistrust within the relationship prompted one of the partners to seek STI testing. This interdependence can be described as syndemic. Notably, the most adversely affected DAS subscale was DS, indicating that couples with an STI experience a tangible reduction in satisfaction with their relationship and a diminished commitment to its continuity. Put differently, low DS scores serve as an indicator of the termination of a relationship. Following DS, AE was the next most affected subscale, showing a decreased expression of affection and sexuality in the couple, likely attributable to heightened irritability, irascibility, reticence, as well as organic symptoms such as genital discharge, bleeding, or itching.

Both the raw and T scores derived from the STAI scale demonstrate an elevation in the STI suspected group compared to the control group. It is plausible that the suspicion of having an STI contributes to a heightened level of transient anxiety during testing. Conversely, the presence of anxiety symptoms at a specific moment may also lead individuals to seek additional medical tests.

There is a lack of literature on adaptation in couples dealing with STIs, except for data on adaptation in couples where at least one partner is affected by HIV infection. A study reveals deficiencies in the adaptation of seronegative partners, particularly in terms of cohesion (DH) [24]. Another investigation indicates that in couples with seropositive HIV status in one partner and seronegative status in the other, there are diminished scores in DS and AE subscales of the DAS. In couples where both partners are HIV-positive, the DAS indicated average scores across all four domains of dyadic adjustment [25].

In a study assessing women aged 13–25 with pelvic inflammatory disease, at three months post-diagnosis, only 25% reported using condoms at their last sexual encounter, and 55% were still in a relationship with the same partner since the time of diagnosis. No association was found between maintaining the same sexual partner and the diagnosis of an STI (OR 0.5; 95% CI: 0.27–1.96). Most young women suffering from pelvic inflammatory disease report stable, exclusive relationships. Given the brief duration of some relationships, couples therapy represents an underexplored opportunity for preventing recurrent STIs [26]. The existing medical literature encompasses only studies focused on the STAI scale in the context of patients infected with HIV. Individuals diagnosed with HIV demonstrate elevated anxiety scores compared to healthy controls [27,28,29]. There are data suggesting that in England, anxiety may predispose individuals to engage in unprotected sexual contact with two or more partners or to acquire sexually transmitted infections in the last year [30]. Besides the anxiety generated by the diagnosis of an STI, patients can show higher stress scores given the fact that they feel stigmatized by the medical staff, family, friends, and acquaintances, they can have insufficient medical insurance coverage, or they could develop certain complications and sequelae of the disease [31,32,33]. Considering that cognitive-behavioral therapy and motivational interviewing-based behavioral interventions could address anxiety symptoms of patients with STI [34], it is important to study the psychological profile of patients who can benefit from these interventions and include interventions in the medical services where patients seek help or on digital platforms [35].

The study group showed a higher number of female participants. This reflects the pattern of patient turnout for STI testing observed in our research. A beneficial aspect was the similarity in gender distribution across both groups under comparison, which supports their comparability. The count of participants identifying as homosexual was limited. This may reflect the considerable stigma associated with this subject in Romania, leading some individuals to withhold their true orientation or categorize themselves under the “others” option instead. By revealing this behavior, this study holds the potential to aid in mitigating the stigma against homosexuals by shedding light on such disparities and encouraging more open discussions and understanding.

In this section, we will discuss the study’s strengths and limitations. This study stands out due to its prospective design, robust statistical analyses, and comprehensive psychological assessments using the Dyadic Adjustment Scale and the State-Trait Anxiety Inventory. With a significant sample size drawn from a geographically diverse region, it offers a detailed examination of the mental health impact of STIs beyond HIV. The study’s focus on the effects of STIs on both individual mental health and dyadic relationships, alongside the use of a control group for comparison, fills a critical gap in sexual health research. It is the first investigation to assess both the quality of relationships and the anxiety provoked by the suspicion of a bacterial STI. These elements collectively enhance the study’s validity, relevance, and potential for broad applicability in understanding the psychological dimensions of STIs. The study encountered limitations as well, notably the reluctance of partners with STIs to participate in the completion of the DAS scale. This posed a challenge in thoroughly analyzing the psychological profile of couples, introducing a constraint in the comprehensive evaluation of relational dynamics. One limitation of this study arises from the observed lack of homogeneity between the defined groups, an issue frequently encountered in the design of control groups for such investigations. Despite efforts to have similar participants on several key demographic and geographic variables, inherent differences in the composition or characteristics of the study and control groups may have influenced the findings. The observational design of the study precludes firm causality inference. Moreover, confounding is a possibility using this design. Nevertheless, we used multivariate models, adjusting for important confounders.

These results not only underscore the psychological burden associated with STIs but also emphasize the necessity for integrated mental health support in the treatment and care of individuals with STIs. Where such support mechanisms are insufficient or absent, the development of referral systems to specialized mental health services could be crucial. Establishing partnerships with mental health professionals and organizations could also facilitate access to necessary support, ensuring that individuals receive comprehensive care that addresses both their physical and emotional well-being. Other measures could involve training healthcare providers in communication skills that are sensitive to the psychological needs of patients facing the stress of potential STI diagnoses.

STAI elevated scores hold significant medical implications, particularly within the public healthcare system, where anxious patients, with potentially confounding anxiety symptoms with manifestations of an organic disease or exhibiting heightened health concerns, may seek numerous medical consultations or tests, resulting in substantial costs for the healthcare system. Therefore, the anxiety experienced by patients with STIs warrants careful consideration and targeted intervention.

## 5. Conclusions

In conclusion, our prospectively designed study underscores the significant mental health impact of STIs, evidenced by lower DAS scores and higher STAI Y-1 scores in individuals with STI suspicion. This observation was particularly pronounced and statistically significant in the total DAS DA score, as well as in the DAS DS and AE subscales. Notably, the association between DAS scores and STIs remained significant after adjusting for confounders in multivariate analyses. Similarly, STAI scores were significantly higher in the STI suspected group, with this association persisting even after controlling for potential confounders.

These findings underscore the necessity for healthcare professionals to adopt a holistic approach to managing patients with STIs, considering not only the physical but also the psychological dimensions of care. Moreover, our results prompt a reevaluation of the current public health policies to incorporate mental health services as a fundamental component of sexual health programs.

## Figures and Tables

**Table 1:** Sexually transmitted infections compared to control group characteristics.

Groups	Control (*n* = 197)	STI Suspected (*n* = 244)	*p*
Age (years), median (IQR)	22 (20–35)	36 (29–43)	<0.001
Sex (f), no. (%)	157 (79.7)	203 (83.2)	0.392
Environment of origin (r), no. (%)	89 (45.18)	104 (42.62)	0.617
STI antecedents, no. (%)	6 (3.05)	28 (12.02)	<0.001
Sexual orientations, no. (%)			0.392
heterosexual:	179 (90.86)	215 (92.27)	
homosexual:	2 (1.02)	0 (0)	
others:	16 (8.12)	18 (7.73)	
Partner with STI, no. (%)	6 (3.05)	26 (11.16)	0.001
More than 2 sexual partners, no. (%)	81 (41.12)	113 (48.92)	0.117
More than 2 sexual partners during the pandemic, no. (%)	12 (6.09)	33 (14.29)	0.006
New sexual partner, no. (%)	9 (4.57)	35 (14.46)	<0.001
Simultaneous sexual partners, no. (%)	4 (2.05)	10 (4.33)	0.189
Age at sexual debut (years), median (IQR)	18 (17–19)	18 (17–19)	0.942
Means of protection against STI, no. (%)			0.003
yes, all the time:	50 (25.38)	32 (13.17)	
occasionally:	49 (24.87)	73 (30.04)	
no:	96 (48.73)	138 (56.79)	
virgin:	2 (1.02)	0 (0)	
Alcohol, no. (%)	35 (17.77)	48 (20.78)	0.468
Psychoactive substances/drugs, no. (%)	0 (0)	5 (2.16)	0.059
Smoking, no. (%)	58 (29.44)	85 (36.8)	0.129
Symptoms			
Dysuria, no. (%)	9 (4.57)	58 (25.11)	<0.001
Pain in the lower abdomen, no. (%)	21 (10.66)	78 (33.77)	<0.001
Dyspareunia, no. (%)	11 (5.58)	61 (26.41)	<0.001
Genital discharge, no. (%)	32 (16.24)	93 (40.26)	<0.001
Metrorrhagia, no. (%)	8 (4.06)	23 (9.96)	0.03
Genital itching, no. (%)	4 (2.03)	31 (13.48)	<0.001
Comorbidities			
Pelvic inflammatory disease, no. (%)	2 (1.02)	13 (5.63)	0.012
Cervicitis/salpingitis/endometritis, no. (%)	2 (1.02)	12 (5.19)	0.022
HPV infection, no. (%)	1 (0.51)	11 (4.72)	0.016
Sterility, no. (%)	2 (1.02)	12 (5.19)	0.026
History of ectopic pregnancies, no. (%)	4 (2.03)	1 (0.43)	0.183
Prostatitis/epididimitis, no. (%)	2 (1.02)	6 (2.6)	0.294
Artritis, no. (%)	3 (1.52)	5 (2.16)	0.746

IQR, interquartile range; STI, sexually transmitted infections; HPV, human papilloma virus.

**Table 2:** Dyadic adjustment scale and state-trait anxiety inventory comparison between sexually transmitted disease group and control group.

Variables	STI Suspected (*n* = 244)	Control (*n* = 197)	Difference (95% CI)	*p*
DAS DC, median (IQR)	57 (48.75–64)	58 (51–64)	−1 (−4; 2)	0.517
DAS DS, median (IQR)	51 (41–58)	56 (48–60)	−5 (−7; −1)	0.006
DAS AE, median (IQR)	50 (40–56)	53 (45–61)	−3 (−5; −0.00003)	0.016
DAS DH, median (IQR)	54 (46–65)	57 (49–65)	−3 (−5; 0.00029)	0.105
DAS DA, median (IQR)	54 (45–63)	58 (49–63.25)	−4 (−6; −0.00004)	0.031
DAS DA category				0.025
Moderately atypical (indicates a significant problem):	13 (10.16)	6 (5.17)		
Mildly atypical (indicates a significant problem):	9 (7.03)	3 (2.59)		
Slightly atypical (borderline: possible reason for concern):	9 (7.03)	2 (1.72)		
Average (normal scores, no reason for concern):	39 (30.47)	36 (31.03)		
Slightly atypical:	23 (17.97)	24 (20.69)		
Mildly atypical:	12 (9.38)	26 (22.41)		
Moderately atypical:	17 (13.28)	15 (12.93)		
Markedly atypical:	6 (4.69)	4 (3.45)		
Moderately/mildly/slightly atypical, no. (%)	31 (24.22)	11 (9.48)		0.002
STAI score, median (IQR)	40 (31–50)	33 (28–40)	7 (3–8)	<0.001
STAI T score, median (IQR)	56 (46–66)	48 (44–56)	8 (4–8)	<0.001

STI, sexually transmitted infections; DAS, dyadic adjustment scale; DC, dyadic consensus; DS, dyadic satisfaction; AE, affectional expression; DH, dyadic cohesion; DA, dyadic adaptability; STAI, state-trait anxiety inventory; IQR, interquartile range; CI, confidence interval; For DAS STI n1 = 128, Control n2 = 116, for STAI STI n1 = 231, Control n2 = 197.

**Table 3:** Multivariate logistic regression models predicting atypical DAS DA (moderately/mildly/slightly atypical) based on STI and adjusted for age, sex, alcohol consumption, psychoactive substance use, and smoking.

Characteristics	OR Adjusted	(95% CI)	*p*
Model 1			
Group (STI suspected vs. control)	2.56	(1.22–5.7)	0.016
Age (years)	1.03	(1–1.06)	0.076
Sex (m vs. f)	0.67	(0.23–1.71)	0.428
Toxic substance consumption	1.19	(0.58–2.46)	0.628
Model 2			
Group (STI vs. control)	2.6	(1.23–5.85)	0.016
Age (years)	1.03	(1–1.06)	0.091
Sex (m vs. f)	0.57	(0.18–1.52)	0.285
Alcohol	1.97	(0.81–4.66)	0.127
Psychoactive substances/drugs	2.45	(0.29–16.57)	0.357
Smoking	0.71	(0.31–1.52)	0.385

DAS, dyadic adjustment scale; DA, dyadic adaptability; OR, odds ratio; CI, confidence interval; STI, sexually transmitted infections.

**Table 4:** Multiple linear regression model predicting STAI T score based on STI and adjusted for age, sex, place of residence, alcohol consumption, psychoactive substance use, smoking, and income source.

Characteristics	B	(95% CI)	*p*
(Intercept)	59.62	(49.8–69.45)	<0.001
Group (STI suspected vs. control)	3.32	(0.5–6.14)	0.021
Age (years)	0	(−0.11–0.11)	0.99
Sex (male vs. female)	−8.38	(−11.48–−5.27)	<0.001
Environment of origin (urban vs. rural)	−0.89	(−3.16–1.38)	0.442
Alcohol	4.18	(1.07–7.29)	0.009
Psychoactive substances/drugs	3.05	(−7.44–13.54)	0.568
Smoking	2.02	(−0.4–4.44)	0.102
Income source (other categories vs. social assistance)	−3.68	(−14.47–7.11)	0.503
Income source (pupil/student vs. social assistance)	−8.43	(−17.34–0.47)	0.063
Income source (no occupation vs. social assistance)	−8.57	(−17.93–0.8)	0.073
Income source (self-employed vs. social assistance)	−5.14	(−14.68–4.41)	0.291
Income source (pensioner/veteran/revolutionary/disabled person vs. social assistance)	−1.47	(−10.91–7.96)	0.759
Income source (employee vs. social assistance)	−7.5	(−15.74–0.74)	0.074
Income source (unemployment vs. social assistance)	3.1	(−21.04–27.23)	0.801

STAI T, State-trait anxiety inventory; CI, confidence interval; STI, sexually transmitted infections.

## Data Availability

The data presented in this study are available upon request from the corresponding author.

## References

[1] Hlatshwayo M., Reno H.E.L., Yarbrough M.L. (2019). STI update: Testing, treatment, and emerging threats. Cleve Clin. J. Med..

[2] Mohammed H., Hughes G., Fenton K.A. (2016). Surveillance systems for sexually transmitted infections: A global review. Curr. Opin. Infect. Dis..

[3] Rogstad K.E. (2019). Sexually transmitted infections and travel. Curr. Opin. Infect. Dis..

[4] Unemo M., Bradshaw C.S., Hocking J.S., de Vries H.J.C., Francis S.C., Mabey D., Fairley C.K. (2017). Sexually transmitted infections: Challenges ahead. Lancet Infect. Dis..

[5] Traeger M.W., Schroeder S.E., Wright E.J., Hellard M.E., Cornelisse V.J., Doyle J.S., Stoové M.A. (2018). Effects of Pre-exposure Prophylaxis for the Prevention of Human Immunodeficiency Virus Infection on Sexual Risk Behavior in Men Who Have Sex With Men: A Systematic Review and Meta-analysis. Clin. Infect. Dis. Off. Publ. Infect. Dis. Soc. Am..

[6] Rowley J., Vander Hoorn S., Korenromp E., Low N., Unemo M., Abu-Raddad L.J., Taylor M.M. (2019). Chlamydia, gonorrhoea, trichomoniasis and syphilis: Global prevalence and incidence estimates, 2016. Bull. World Health Organ..

[7] National Academies of Sciences, Engineering, and Medicine (2021). Sexually Transmitted Infections: Adopting a Sexual Health Paradigm.

[8] Weinstock H.S., Kreisel K.M., Spicknall I.H., Chesson H.W., Miller W.C. (2021). STI Prevalence, Incidence, and Costs in the United States: New Estimates, New Approach. Sex. Transm. Dis..

[9] John S.A., Walsh J.L., Cho Y.I., Weinhardt L.S. (2018). Perceived Risk of Intimate Partner Violence Among STI Clinic Patients: Implications for Partner Notification and Patient-Delivered Partner Therapy. Arch. Sex. Behav..

[10] Passaro R.C., Segura E.R., Gonzales-Saavedra W., Lake J.E., Perez-Brumer A., Shoptaw S., Clark J.L. (2020). Sexual Partnership-Level Correlates of Intimate Partner Violence Among Men Who Have Sex with Men and Transgender Women in Lima, Peru. Arch. Sex. Behav..

[11] Karamouzian M., Knight R., Davis W.M., Gilbert M., Shoveller J. (2018). Stigma associated with sexually transmissible infection testing in an online testing environment: Examining the perspectives of youth in Vancouver, Canada. Sex. Health.

[12] Thomas J.A., Ditchman N., Beedle R.B. (2022). The impact of knowledge, self-efficacy, and stigma on STI testing intention among college students. J. Am. Coll. Health.

[13] Zeidan A.R., Strey K., Vargas M.N., Reveles K.R. (2021). Sexually transmitted infection laboratory testing and education trends in US outpatient physician offices, 2009–2016. Fam. Med. Community Health.

[14] Hart T.A., Noor S.W., Vernon J.R.G., Antony M.M., Gardner S., O’Cleirigh C. (2020). Integrated Cognitive-Behavioral Therapy for Social Anxiety and HIV/STI Prevention for Gay and Bisexual Men: A Pilot Intervention Trial. Behav. Ther..

[15] Covey J., Rosenthal-Stott H.E.S., Howell S.J. (2016). A synthesis of meta-analytic evidence of behavioral interventions to reduce HIV/STIs. J. Behav. Med..

[16] Church K., Mayhew S.H. (2009). Integration of STI and HIV prevention, care, and treatment into family planning services: A review of the literature. Stud. Fam. Plan..

[17] O’Connor E., Lin J.S., Burda B.U., Henderson J.T., Walsh E.S., Whitlock E.P. (2014). Behavioral Sexual Risk Reduction Counseling in Primary Care to Prevent Sexually Transmitted Infections: An Updated Systematic Evidence Review for the U.S. Preventive Services Task Force [Internet].

[18] World Health Organization [Internet]. Europe Targets of Sustainable Developmen Goal 3; [about 2 Screens]. https://www.who.int/europe/about-us/our-work/sustainable-development-goals/targets-of-sustainable-development-goal-3.

[19] The Population and Housing Census 2021. [Internet] Romania. https://www.recensamantromania.ro/rezultate-rpl-2021/rezultate-definitive/.

[20] Grad A.I., Vica M.L., Ungureanu L., Siserman C.V., Tătaru A.D., Matei H.V. (2020). Assessment of STI screening in Romania using a multiplex PCR technique. J. Infect. Dev. Ctries..

[21] Iliescu D., Spanier G.B. (2009). Scala de Adaptare in Cuplu: Manual Tehnic.

[22] Pitariu H., Peleasa C., Spielberger C.D. (2007). State-Trait Anxiety Inventory.

[23] R Core Team (2023). R: A Language and Environment for Statistical Computing.

[24] Martins A., Canavarro M.C., Pereira M. (2021). The relationship between dyadic coping and dyadic adjustment among HIV-serodiscordant couples. AIDS Care.

[25] Largu A., Manciuc C., Vâţă A., Nicolau C., Prisacaru L., Ciubotaru F.F., Dorobat C.M. (2012). Dyadic adjustment in HIV sero-concordant and sero-discordant couples. Rev. Med.-Chir. Soc. Med. Nat. Iasi.

[26] Tabacco L., Chung S.E., Perin J., Huettner S., Butz A., Trent M. (2018). Relationship Status and Sexual Behaviors in Post-Pelvic Inflammatory Disease (PID) Affected Urban Young Women: A Sub-Study of a Randomized Controlled Trial. Int. Arch. Nurs. Health Care.

[27] Zinkernagel C., Taffé P., Rickenbach M., Amiet R., Ledergerber B., Volkart A.C., Swiss H.I.V. (2001). Importance of mental health assessment in HIV-infected outpatients. J. Acquir. Immune Defic. Syndr..

[28] Pakesch G., Pfersmann D., Loimer N., Grünberger J., Linzmayer L., Mayerhofer S. (1992). Noopsychological changes and psychopathological characteristics of HIV-1 patients of various risk groups. Fortschr. Neurol. Psychiatr..

[29] Levy I., Goldstein A., Fischel T., Maor Y., Litachevsky V., Rahav G. (2013). Neurocognitive disturbances and psychiatric disorders among patients living with HIV-1 positive in Israel. Harefuah.

[30] Coyle R.M., Lampe F.C., Miltz A.R., Sewell J., Anderson J., Apea V., Rodger A. (2019). Associations of depression and anxiety symptoms with sexual behaviour in women and heterosexual men attending sexual health clinics: A cross-sectional study. Sex. Transm. Infect..

[31] Young S.D., Nussbaum A.D., Monin B. (2007). Potential moral stigma and reactions to sexually transmitted diseases: Evidence for a disjunction fallacy. Pers. Soc. Psychol. Bull..

[32] Trifu M.P., Vică M., Pintea L., Siserman C., Matei H. (2022). The medical staff’s perception of stigma in sexually transmitted diseases. Rom. J. Leg. Med..

[33] Pintea-Trifu M.L., Bâlici Ş., Siserman C.V., Vică M.L., Matei H.V. (2023). Chlamydia trachomatis and the HLA involvement in the development of infection and disease: A narrative review. Med. Pharm. Rep..

[34] Bretz L.A., Keshwani N., Raphael M. (2023). Relationship between mental health diagnoses and sexually transmitted infections. Bull. Menn. Clin..

[35] Cao B., Bao H., Oppong E., Feng S., Smith K.M., Tucker J.D., Tang W. (2020). Digital health for sexually transmitted infection and HIV services: A global scoping review. Curr. Opin. Infect. Dis..

